# Editorial Note

**DOI:** 10.1002/2211-5463.13073

**Published:** 2021-01-21

An anonymous user of PubPeer reported the presence of duplicated and horizontally flipped western blot bands in Figure 5C of the article ‘The activation mechanism of the aryl hydrocarbon receptor (AhR) by molecular chaperone HSP90’ https://doi.org/10.1016/j.fob.2014.09.003. This duplication was subsequently confirmed by our image integrity analyst. The β‐actin bands for the cytosol sample at 120 min after β‐NF or DMSO treatment are identical to the β‐actin bands for the cytosol sample at 360 min after β‐NF or DMSO treatment, but are horizontally flipped.

The corresponding author informed the journal that the original data for this manuscript have been discarded, and thus it is no longer possible to investigate this apparent error.

This Editorial Note is intended to alert our readers to the fact that the data for the 120 and 360 min time points in Figure 5C should be treated with caution. However, the translocation of AhR from the cytosol to the nucleus in response to β‐NF treatment can still be observed by comparison of the western blots of samples treated for 0, 15, 30, and 60 min. Therefore, we are satisfied that the key conclusions of the manuscript are not affected by the error in this figure.

